# Resveratrol Protects the Brain of Obese Mice from Oxidative Damage

**DOI:** 10.1155/2013/419092

**Published:** 2013-09-15

**Authors:** Shraddha D. Rege, Sruthi Kumar, David N. Wilson, Leslie Tamura, Thangiah Geetha, Suresh T. Mathews, Kevin W. Huggins, Tom L. Broderick, Jeganathan Ramesh Babu

**Affiliations:** ^1^Department of Nutrition, Dietetics, and Hospitality Management, Auburn University, Auburn, AL 36849, USA; ^2^Department of Physiology, Laboratory of Diabetes and Exercise Metabolism, Midwestern University, Glendale, AZ 85308, USA; ^3^Department of Physical Sciences, Auburn University at Montgomery, Montgomery, AL 36117, USA

## Abstract

Resveratrol (3,5,4′-trihydroxy-trans-stilbene) is a polyphenolic phytoalexin that exerts cardioprotective, neuroprotective, and antioxidant effects. Recently it has been shown that obesity is associated with an increase in cerebral oxidative stress levels, which may enhance neurodegeneration. The present study evaluates the neuroprotective action of resveratrol in brain of obese (*ob/ob*) mice. Resveratrol was administered orally at the dose of 25 mg kg^−1^ body weight daily for three weeks to lean and obese mice. Resveratrol had no effect on body weight or blood glucose levels in obese mice. Lipid peroxides were significantly increased in brain of obese mice. The enzymatic antioxidants superoxide dismutase, catalase, glutathione peroxidase, glutathione reductase, glucose-6-phosphate dehydrogenase and nonenzymatic antioxidants tocopherol, ascorbic acid, and glutathione were decreased in obese mice brain. Administration of resveratrol decreased lipid peroxide levels and upregulated the antioxidant activities in obese mice brain. Our findings indicate a neuroprotective effect of resveratrol by preventing oxidative damage in brain tissue of obese mice.

## 1. Introduction

Obesity is a major risk factor for the development of type 2 diabetes. Roughly 30 percent of obese people are diabetic, and 85 percent of diabetics are obese. Other obesity-related conditions include heart disease, stroke, and certain types of cancer. According to the National Institutes of Health around 97 million Americans are affected by these conditions which is the second leading cause of death. Recently obesity has been shown to increase the level of cerebrocortical reactive oxygen species and impair brain function [1], suggesting that obesity may increase the risk for neurodegenerative conditions such as Alzheimer's disease [2, 3]. 

Oxidative stress is associated with an increase in oxidizing species that destructs the vascular and neuronal cells in central nervous system. Oxidative stress is due to the imbalance between the oxygen free radicals generated and the antioxidant defense system to detoxify the reactive intermediates [4]. Oxidative stress changes the signaling pathways that may induce cellular responses such as inflammation, cell proliferation, and cell survival and death [5]. Reactive oxygen species (ROS) are chemically reactive molecules that consist of oxygen ions and peroxides that include hydrogen peroxide, singlet oxygen, nitric oxide, peroxynitrite, and superoxide free radicals. The release of peroxides and free radicals is toxic to the cell, which may lead to cell death. The antioxidant enzymes, such as superoxide dismutase (SOD), catalase, and peroxidases, and nonenzymatic free radical scavengers (ascorbic acid, *α*-tocopherol, and GSH) convert the reactive oxygen species to water and oxygen, the stable molecules. These antioxidants are known to protect the cells and tissues against oxidative injury caused by reactive oxygen species [6]. Obesity has found to increase the levels of total reactive oxygen species in brain, thereby increasing susceptibility to oxidative stress and neurodegeneration [1]. 

Resveratrol (3,5,4′-trihydroxystilbene) a naturally occurring polyphenol belonging to the phytoalexin family is found in peanuts [7], skin, and seeds of grapes [8]. Evidence indicates that resveratrol exerts neuroprotective effects against diabetes-induced oxidative damage [9, 10]. Resveratrol is also cardioprotective [11], anti-inflammatory [12] prevents certain cancers [13] and improves insulin sensitivity in diet-induced obese mice [14]. The present study was designed to evaluate the neuroprotective action of resveratrol on obese (*ob/ob*) mice induced oxidative stress.

## 2. Materials and Methods

### 2.1. Reagents

Resveratrol (*trans*) was purchased from Sigma Chemical Co. (St. Louis, MO, USA). All other chemicals and solvents were of analytical grade and were obtained from Sigma Chemical Co. (St. Louis, MO, USA).

### 2.2. Animals

This study was approved by the Midwestern University Research and Animal Care Committee. Male 8-week-old B6.V-Lep/J *ob/ob* mice were obtained from Jackson Laboratory (Bar Harbor, Maine). The *ob/ob* mouse was selected because it exhibits metabolic abnormalities including hyperglycemia and hyperinsulinemia that phenotypically resembles human type 2 diabetes and severe obesity. Age-matched C57BL/J6 mice were used as lean controls. Mice were housed 2 per cage and were provided with food and water provided *ad libitum*, maintained in a room with alternating twelve hour light/dark cycle, and kept at 22°C. Mice were maintained on a standard pellet diet (LabDiet 5001, PMI Nutrition International, Inc., Brentwood, MO, USA) for 21 days, including the 24 hour period of when food intake was measured. The composition of the diet (based on chemical composition) according to the manufacturer was as follows: 23.9% protein, 10.7% fat, 5.1 fiber, 48.7% carbohydrate, 7% mineral mixture, and 4.6% vitamin mixture.

### 2.3. Experimental Design

After one week of acclimatization, obese and lean control mice were divided into four groups consisting of six animals each: lean control, lean control-resveratrol treated, obese, and obese-resveratrol treated. Trans-resveratrol was mixed with a 1% solution of methylcellulose (Sigma-Aldrich, MO, USA), with viscosity 25 cP to form a colloid, which was administered by oral gavage in volume of 0.5 to 0.75 mL at a concentration of 25 mg/kg body weight once daily for a period of 21 days. Lean mice received the vehicle only. This methylcellulose formulation was well tolerated by mice, and there was no evidence of gastrointestinal distress, changes in behavior and ambulatory activity, or dramatic weight loss in mice. This concentration of resveratrol and duration of treatment were selected based on previous studies highlighting its insulin-mimetic properties, beneficial effects on cardiac and endothelial function, and anti-inflammatory and neuroprotective effects [15–18]. Recent evidence also indicates that significant levels of resveratrol are detected in rat brain following 3 daily doses of 25 mg/kg of trans-resveratrol [18], despite the short half-life and bioavailability of this conjugated form in plasma [19].

### 2.4. Food Intake

Mice from each group were placed individually in metabolic cage systems (Mini Mitter, Bend, OR) between 8:00 and 9:00 am during the final week of study for a 24-hour period for measurement of food intake. 

### 2.5. Blood and Tissue Sampling

After the 3-week treatment period, mice were sacrificed in the morning between 10 am and 1 pm. Following CO_2_ asphyxiation, a sternotomy was performed to expose the heart, and then blood was obtained by cardiac puncture from the right ventricle. Blood was centrifuged (3,500 rpm at 4°C, for 5 min), and plasma was separated from the erythrocytes for the assay of glucose using a commercially available kit (Wako Chemical, VA). Brain tissues were excised immediately, and a 10% homogenate was prepared in 100 mM Tris HCl (pH 7.4) using a Potter-Elvehjem homogeniser and used for the estimation of biochemical parameters. Adipose tissue was dissected and then weighed.

### 2.6. Estimation of Protein

The amount of protein in the tissue homogenate was measured by the method of Pierce using BSA as standard. 150 *μ*L of protein assay reagent was added to 10 *μ*L of the brain tissue homogenate, and the color developed was read after 5 min at 630 nm. The levels of protein are expressed as mg/mL.

### 2.7. Lipid Peroxidation

Lipid peroxidation was estimated in brain tissue homogenate by the method of Hogberg et al. [20] using thiobarbituric acid. The release of malondialdehyde as an end product of peroxidation of lipids served as the index of the intensity of oxidative stress. Given the limitation of this method, direct measurement of lipid hydroperoxide was also carried out using a commercial kit (Cayman Chemical Co., Ann Arbor, MI, USA).

### 2.8. Enzymic Antioxidants

Antioxidant enzymes were estimated in brain tissue homogenate of experimental groups. Superoxide dismutase (SOD) isoforms SOD1 and SOD2 were assayed using the kit (Cayman Chemical Co., Ann Arbor, MI, USA) according to the manufacturers' standard procedures. Catalase activity was assayed by the method of Sinha [21]. Glutathione peroxidase (GPX) was assayed by the method of Rotruck et al. [22]. The utilization of glutathione was used to express the activity. Glutathione reductase (GR), that utilizes NADPH to convert oxidized glutathione (GSSG) to the reduced form was measured by the method of Staal et al. [23]. The activity of glucose-6-phosphate dehydrogenase (G6PDH) was assayed by the method of Ells and Kirkman [24].

### 2.9. Nonenzymatic Antioxidants

Ascorbic acid is oxidized by copper to form dehydroascorbic acid and diketogulonic acid. Dehydroascorbic acid reacts with 2,3-dinitrophenyl hydrazine to form the derivative of 2,4-dinitrophenyl hydrazine. This compound in strong sulphuric acid undergoes a rearrangement to form a product with absorption maxima at 520 nm. The reaction was run in the presence of thiouria to prevent the interference of non-ascorbic acid chromogens [25]. *α*-Tocopherol was estimated by the method of Quaife et al. [26]; the reduced glutathione levels (GSH) and oxidized glutathione (GSSG) were quantified according to kit manufacturer's instructions (Cayman Chemical Co., Ann Arbor, MI, USA). The redox index was calculated as (GSH = 2GSSG)/(2GSSG × 100) reported by Öztürk and Gümüşlü [27].

### 2.10. Statistical Analysis

All values are expressed as mean ± standard deviation (S.D.) in each group. Statistical difference between groups was assessed by one-way ANOVA followed by Tukey-Kramer analyses with equal variance. Significance was set at *P* < 0.05.

## 3. Results

### 3.1. Resveratrol Had No Effect on Body Weight of Obese Mice

The effects of resveratrol treatment on physical characteristics of obese mice are illustrated in Table 1(a). Confirming the phenotype of the *ob/ob* mouse, body weight and fat pad weight were significantly greater (*P* < 0.001) in obese mice compared to lean control mice. However, body weight was not altered with resveratrol treatment of obese mice. There was no significant difference in the fat pad weight between obese and obese-resveratrol treated groups, although fat pad weight tended to be lower by ~15% in obese mice treated with resveratrol.

### 3.2. Resveratrol Did Not Reduce the Blood Glucose in Obese Mice

Blood glucose levels in lean and obese mice after resveratrol treatment are shown in Table 1(b). Also confirming the characteristics of the *ob/ob* mouse, blood glucose levels were significantly elevated (*P* < 0.001) in obese mice compared to lean control mice. Treatment with resveratrol had no effect on plasma glucose levels in both lean and obese mice. Intriguingly, in obese mice, blood glucose levels were increased by ~19% following resveratrol treatment.

Food intake in lean and obese mice with and without resveratrol treatment is illustrated in Table 1(b). Resveratrol had no significant effect on food intake in both lean and obese mice. However, food intake was highest (*P* < 0.05) in the obese-resveratrol treated group compared to lean groups.

### 3.3. Resveratrol Ameliorated Lipid Peroxidation Induced in Obese Mice

**Figure 1:**
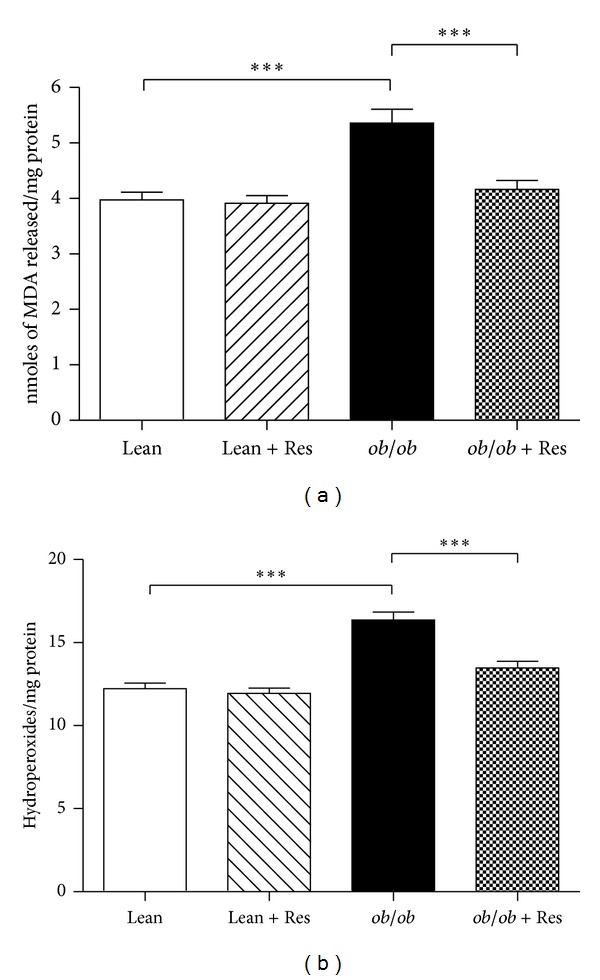
Effect of resveratrol on the levels of malondialdehyde (a) and hydroperoxide (b) in lean and obese mice brains. Values are expressed as mean ± S.D. for six mice in each group. Values are statistically significant at ***P < 0.001.

Figure 1 shows the effect of resveratrol on malondialdehyde (Figure 1(a)) and lipid peroxides (Figure 1(b)) levels in brain of the lean and obese mice. The levels were substantially increased (*P* < 0.001) in obese mice brain compared to lean control mice. Administration of resveratrol for 3 weeks to obese mice significantly reduced (*P* < 0.001) the lipid peroxidation when compared to obese untreated mice.

### 3.4. Resveratrol Improves the Enzymic Antioxidants in Obese Mice

Table 2 presents the levels of antioxidant enzymes in lean and obese mice with or without resveratrol administration. We found that in obese control mice brain the activities of antioxidant enzymes: SOD, catalase, GPX, GR, and G6PD were significantly declined (*P* < 0.001) when compared to lean control mice. Resveratrol administration to the obese mice enhanced (*P* < 0.001) the enzymic antioxidant activities in brain to a significant extent compared to obese control mice. However resveratrol administration did not show any significant change in SOD2 activity. There were no significant changes in the levels of enzymic antioxidants of lean mice administered resveratrol in comparison to untreated lean mice.

### 3.5. Resveratrol Enhanced the Nonenzymatic Antioxidants in Obese Mice

The nonenzymatic antioxidants were reduced in obese mice brains to the same extent as those observed for the enzymatic antioxidants (Table 3). The levels of ascorbic acid, *α*-tocopherol, GSH, and GSH/GSSG ratio were significantly decreased in obese control mice brain compared with lean control mice. Resveratrol administered to obese mice enhanced the levels of ascorbic acid (*P* < 0.001), *α*-tocopherol (*P* < 0.05), and GSH (*P* < 0.01) levels compared to obese-untreated mice. There were no marked changes observed in resveratrol treated lean mice.

## 4. Discussion

Oxidative stress leads to neurodegeneration due to insufficiency of the antioxidant defense mechanisms in the brain to counteract the increased reactive oxygen species formation [28–31]. Dietary supplements containing antioxidants might be favorable in maintaining the brain function [32]. For instance, in PC12 cells, the antioxidant resveratrol has found to be neuroprotective against oxidative stress [33], by attenuating the generation of free radicals. Evidence also indicates that resveratrol suppresses oxidative stress-induced neuronal cell death [34] and blocks lipid peroxidation [35]. Resveratrol has been shown to improve the memory loss and protect the rats from A*β*-induced neurotoxicity by reducing the inducible nitric oxide synthase and lipid peroxides [36]. The present study was designed to examine the salubrious effects of resveratrol as a neuroprotective antioxidant on brain of *ob/ob* mice, a model of severe obesity with insulin resistance, resulting from defective leptin signaling. We chose the representative concentration of resveratrol of 25 mg/kg based on the observations that this polyphenol improves the overall diabetic state and accumulates in tissues, including heart, liver, kidney, and brain, following acute and chronic treatments [15, 18, 37–39]. We demonstrate that obesity has a negative impact on the oxidative stress in the central nervous system of *ob/ob* mice. Indeed, brain from obese mice exhibits increased lipid perioxidation, along with a decrease in the levels of key neuroprotective antioxidants. Resveratrol was clearly beneficial by reversing lipid peroxidation and improving the antioxidant status.

In brain, polyunsaturated fatty acids exposed to reactive oxygen species result in the production of toxic lipid peroxidation intermediates [40]. A significant increase in lipid peroxidation was observed in brain of *ob/ob* mice, confirming earlier reports [1]. Recent evidence indicates that a diet high in fat was found to increase the level of lipid peroxidation in rat brain [41]. Lipid peroxidation is also increased in brain of streptozotocin-induced diabetic rats, suggesting that lipid peroxidation is not solely related to defective leptin signaling and hyperglycemia [9]. Administration of resveratrol reduced the lipid peroxidation level of the obese mice significantly. The beneficial effect of resveratrol on lipid peroxidation in brain was also reported in streptozotocin-induced diabetic rats [9]. 

The cellular antioxidant defense mechanism against reactive oxygen species includes enzymatic defense systems such as SOD, catalase, and GPX. SOD converts the superoxide radical to H_2_O_2_, which, in turn, is further eliminated by catalase and GPX. The activities of these enzymes are reduced in *ob/ob* mice compared to lean mice. Mice fed with high fat diet demonstrated a significant decrease in GPX activity in cortex but no difference in SOD in cortex and hippocampus [1]. Obesity increases the total reactive oxygen species and superoxide in brain [1], which might explain the decreased activities of SOD, catalase, and GPX observed in obese mice. In brain from obese mice, catalase activity might be reduced due to reduced NADPH levels, since the regeneration of catalase from its inactive form requires NADPH. GPX activity is also reduced in brain from obese mice due to reduction of GSH levels and its inactivation by the accumulation of superoxide radicals. In this study, we are the first to report that administration of resveratrol significantly increased the enzymic antioxidant activities in obese mice. 

In addition to enzymic antioxidants, nonenzymatic antioxidants also protect the brain from oxidative damage. GSH is an endogenous nonenzymatic antioxidant against reactive oxygen species in the cellular defense system. GSH is oxidized to glutathione disulfide (GSSG) by reactive oxygen species, thereby reducing the level of GSH. Glutathione reductase (GR) converts GSSG back to GSH by NADPH, which in turn is released by glucose-6-phosphate dehydrogenase (G6PDH). The level of these antioxidants is reduced in both obese mice and diabetes mellitus [42–44]. Administration of resveratrol increased the activities of these antioxidants in obese mice, as well as that of *α*-tocopherol and ascorbic acid, which are also reduced in obese mice brain. Ascorbic acid is a water-soluble antioxidant [45] which prevents the degradation of tocopherol to tocopheroxyl radical [46]. *α*-Tocopherol is a lipophilic antioxidant and functions as a peroxyl radical scavenger. It blocks lipid peroxidation by reacting with free radicals, thereby forming *α*-tocopherol radical, which is then oxidized by ascorbic acid and converted back to its reduced state [47]. In the present study, elevation of ascorbate and *α*-tocopherol was observed in obese mice brain treated with resveratrol. The levels of ascorbate and *α*-tocopherol might be elevated due to increase in GSH activity on resveratrol administration.

Early investigations have addressed the kinetics of absorption and bioavailability of resveratrol and its conjugated forms in serum. It is known that conjugated resveratrol is less biologically active than resveratrol provided or ingested in its natural red wine matrix and that absorption of resveratrol may by altered by other dietary media such as juice homogenates [48, 49]. In diabetic mice, it is possible that elevated brain glucose levels [50], may interfere with accumulation of resveratrol and limit its antioxidant properties. Yet, the treatment regimen used in this study has been linked to several beneficial neuroprotective effects, indicating that trans-resveratrol does accumulate in tissues after short-term treatment, and overcoming the metabolic perturbations associated with hyperglycemia in brain of diabetic rodents [50]. Further, earlier feeding studies in the rat have demonstrated that red wine administered acutely by oral gavage at the much lower dose of 80 *μ*g/kg resulted in a significant accumulation of total resveratrol in heart, liver, and kidney within 30 to 240 minutes. Reducing the dosage by one-half with continued treatment over 15 days also produced dramatic increase in tissue resveratrol content [37]. In this study, bioavailability of trans-resveratrol is clearly not rate limiting with evidence indicating that accumulation of resveratrol in brain is seen following short-term administration of resveratrol at the concentration of 25 mg/kg [18]. 

It was interesting to observe that food intake in *ob/ob* mice treated with resveratrol was elevated compared to nondiabetic mice. Food intake measurements were initiated after the observation that resveratrol decreased body weight in *ob/ob* mice, believing that effect was secondary to perhaps a reduction in food intake. Although administration of resveratrol is linked to a reduction in food intake in diabetic rodents [38], food intake was increased in the *ob/ob* mice compared to nonobese mice, suggesting improvements in overall oxidative metabolism occurring to resveratrol. To our knowledge, a direct central nervous system-mediated hyperphagic response of resveratrol has not been reported in this model of obesity and diabetes. However, there is evidence that resveratrol, by activating the sirtuin system in peripheral tissues, improves glucose metabolism, mitochondrial function, and biogenesis, and may be hormetic in nature [39, 48].

## 5. Conclusions

This study suggests that resveratrol is effective in preventing against obesity-induced oxidative damage in brain. Indeed, in brain of *ob/ob* mice, the reduction in the antioxidative status is attenuated, indicating that resveratrol exerts both antioxidant and neuroprotective properties. Our findings provide the rationale for further studies directed in understanding of mechanism of resveratrol in preventing neurodeterioration.

## Figures and Tables

**Table:** (a)

Parameter	Lean	Lean + Res	*ob/ob *	*ob/ob* + Res
Body weight (g)	25.1 ± 1.58	23.9 ± 1.26	49.3 ± 6.19^a∗∗∗^	47.2 ± 4.50^bNS^
Fat pad (g)	1.24 ± 0.24	1.08 ± 0.11	13.12 ± 3.71^a∗∗∗^	11.23 ± 2.75^bNS^

**Table:** (b)

Parameter	Lean	Lean + Res	*ob/ob *	*ob/ob* + Res
Blood glucose (mg/dL)	124.2 ± 19.40	111.8 ± 21.24	242.2 ± 58.1^a∗∗∗^	288.4 ± 44.4^bNS^
Food intake (g/day)	4.44 ± 0.11	4.96 ± 1.61	6.58 ± 3.24^aNS^	9.56 ± 3.28^c∗^

Values are expressed as mean ± S.D. for twelve mice in each group.

Values are statistically significant at _ _***P < 0.001 and _ _*P < 0.05.

^a^ *ob/ob* mice were compared with lean control mice; ^b^ *ob/ob* mice were compared with *ob/ob*-resveratrol treated mice; ^c^lean mice were compared with *ob/ob*-resveratrol treated mice. NS represents nonsignificant.

**Table 2:** Effect of resveratrol on the levels of enzymic antioxidants in lean and obese mice brains.

Parameter	Lean	Lean + Res	*ob/ob *	*ob/ob* + Res
SOD1	71.80 ± 3.82	72.62 ± 3.53	46.96 ± 3.56^a∗∗∗^	63.75 ± 3.26^b∗∗∗^
SOD2	80.11 ± 4.93	80.61 ± 5.14	71.41 ± 4.43^a∗∗^	77.15 ± 4.74^bNS^
Catalase	6.93 ± 0.49	6.95 ± 0.61	4.07 ± 0.37^a∗∗∗^	6.07 ± 0.49^b∗∗∗^
GPx	7.44 ± 0.99	7.66 ± 0.84	4.37 ± 0.52^a∗∗∗^	6.60 ± 0.59^b∗∗∗^
GR	0.42 ± 0.04	0.43 ± 0.03	0.19 ± 0.02^a∗∗∗^	0.38 ± 0.03^b∗∗∗^
G6PD	571.2 ± 28.6	586.1 ± 23.9	292.9 ± 20.4^a∗∗∗^	496.9 ± 19.2^b∗∗∗^

Values are expresses as mean ± S.D. for six mice in each group. SOD: amount of enzyme required to exhibit 50% dismutation of superoxide radical/mg protein; catalase: nmoles of H_2_O_2_ consumed/min/mg protein; GPX: mmoles of GSH oxidized/min/mg protein; GR: nmoles of NADPH consumed/min/mg protein; G6PD: nmoles of NADPH liberated/min/mg protein. Values are statistically significant at **P < 0.01 and ***P < 0.001. ^a^ *ob/ob *mice were compared with lean control mice; ^b^ *ob/ob *mice were compared with *ob/ob*-resveratrol treated mice. NS represents nonsignificant.

**Table 3:** Effect of resveratrol on the levels of nonenzymic antioxidants in lean and obese mice brains.

Parameter	Lean	Lean + Res	*ob/ob *	*ob/ob* + Res
Ascorbic acid	1.57 ± 0.20	1.51 ± 0.21	0.76 ± 0.12^a∗∗∗^	1.32 ± 0.18^b∗∗∗^
*α*-Tocopherol	1.62 ± 0.19	1.58 ± 0.19	1.06 ± 0.20^a∗∗∗^	1.42 ± 0.19^b∗^
GSH	4.73 ± 0.60	5.01 ± 0.43	3.77 ± 0.31^a∗∗^	4.44 ± 0.37^b∗∗^
GSSG	1.71 ± 0.20	1.88 ± 0.24	1.65 ± 0.20^aNS^	1.73 ± 0.19^bNS^
GSH/GSSG	2.69 ± 0.16	2.74 ± 0.19	2.30 ± 0.12^a∗∗∗^	2.57 ± 0.11^b∗∗^
Redox index	0.023 ± 0.001	0.024 ± 0.001	0.021 ± 0.001^a∗∗∗^	0.023 ± 0.001^b∗∗^

Values are expresses as mean ± S.D. for six mice in each group. Ascorbic acid, mg/mg protein; *α*-tocopherol: mg/mg protein; GSH and GSSG: nmol/mg protein. Values are statistically significant at ***P < 0.001, **P < 0.01, and *P < 0.05. ^a^ *ob/ob *mice were compared with lean control mice; ^b^ *ob/ob *mice were compared with *ob/ob*-resveratrol treated mice. NS represents nonsignificant.

## Abbreviations

*ob/ob*: Obese
ROS: Reactive oxygen species
LPO: Lipid peroxidation
MDA: Malondialdehyde
SOD: Superoxide dismutase
GPX: Glutathione peroxidase
GR: Glutathione reductase
G6PDH: Glucose-6-phosphate
GSH: Glutathione.
